# Genome-Wide Analysis of the Expression of *WRKY* Family Genes in Different Developmental Stages of Wild Strawberry (*Fragaria vesca*) Fruit

**DOI:** 10.1371/journal.pone.0154312

**Published:** 2016-05-03

**Authors:** Heying Zhou, Yuxuan Li, Qing Zhang, Suyue Ren, Yuanyue Shen, Ling Qin, Yu Xing

**Affiliations:** 1 College of Plant Science and Technology, Beijing University of Agriculture, Beijing, 102206, PR China; 2 Beijing Key Laboratory for Agricultural Application and New Technique, Beijing University of Agriculture, Beijing,102206, PR China; Key Laboratory of Horticultural Plant Biology (MOE), CHINA

## Abstract

WRKY proteins play important regulatory roles in plant developmental processes such as senescence, trichome initiation and embryo morphogenesis. In strawberry, only *FaWRKY1* (*Fragaria × ananassa*) has been characterized, leaving numerous WRKY genes to be identified and their function characterized. The publication of the draft genome sequence of the strawberry genome allowed us to conduct a genome-wide search for WRKY proteins in *Fragaria vesca*, and to compare the identified proteins with their homologs in model plants. Fifty-nine *FvWRKY* genes were identified and annotated from the *F*. *vesca* genome. Detailed analysis, including gene classification, annotation, phylogenetic evaluation, conserved motif determination and expression profiling, based on RNA-seq data, were performed on all members of the family. Additionally, the expression patterns of the *WRKY* genes in different fruit developmental stages were further investigated using qRT-PCR, to provide a foundation for further comparative genomics and functional studies of this important class of transcriptional regulators in strawberry.

## Introduction

Members of the WRKY class of transcription factors, which are ubiquitous among higher plants, exhibit sequence-specific DNA-binding and are capable of activating or repressing the transcription of downstream target genes [1]. Proteins in this superfamily contain either one or two highly conserved signature domains of approximately 60 amino acid residues, including the conserved WRKYGQK sequence followed by a zinc finger structure in the C-terminal region [2]. Studies have also shown that the conserved WRKY domain can have slightly longer sequences, such as WRKYGKK and WEKYGQK [3], or that it can be replaced by WKKY, WKRY, WSKY, WIKY, WRIC, WRMC, WRRY or WVKY. The conservation of the WRKY domain is mirrored by a remarkable conservation of its cognate binding site, the W box (TTGACC/T) [4–6]. The WRKY domain facilitates binding of the protein to the W box or the SURE (sugar-responsive *cis*-element) element in the promoter regions of target genes [7, 8]. As described by Eulgem et al. (2000), WRKY proteins can be divided into three major groups based on both the number of WRKY domains and the specific features of their zinc-finger-like motif: WRKY proteins with two WRKY domains containing a C_2_H_2_ zinc-finger motif belong to group I, whereas most proteins with one WRKY domain containing a C_2_H_2_ zinc-finger motif belong to group II, which can be further divided into five subgroups (IIa, IIb, IIc, IId and IIe). Generally, the same type of finger motif is characteristic for group I and group II members (C-X_4-5_-C-X_22-23_-H-X_1_-H,). Group III consists of a small number of genes characterized by a single WRKY domain with a C_2_HC zinc-finger motif.

Since the first cDNA encoding a WRKY protein, *SPF1*, was cloned from sweet potato (*Ipomoea batatas*) [9], numerous members of the family have been characterized from several plant species, and they have been found to be involved in various physiological processes under normal growth conditions and under various biotic and abiotic stresses [10–20]. There are at least 72 WRKY family members in *Arabidopsis thaliana* and at least 109 in rice (*Orza sativa*). Furthermore, 55 *WRKY* genes have been identified in the cucumber (*Cucumis sativus*) genome and 59 putative grapevine (*Vitis vinefera*) WRKY transcription factors were also identified following a search of various genomic and proteomic grapevine databases [21,22]. Many of the *A*. *thaliana* WRKY proteins appear to be involved in regulating the balance between salicylic acid (SA)- and jasmonic acid (JA)-dependent defense pathways. One example is AtWRKY70, a common regulatory component of SA- and JA-dependent defense signaling, which mediates the cross-talk between these antagonistic pathways and is a positive regulator of R-gene mediated resistance and systemic defense responses [23,24]. Microarray analyses have revealed that expression of some of the *A*. *thaliana* WRKY transcripts is strongly regulated by various abiotic stresses, such as salinity, drought and cold [25,26]. Moreover, abiotic stresses (salinity, drought and cold) and phytohormone treatments were reported to result in changes in the transcript levels of 54 rice *WRKY* genes [20]. Finally, WRKY proteins are also known to play important regulatory roles in developmental processes such as senescence, trichome initiation and embryo morphogenesis [27–30].

While the WRKY family has been well studied in model experimental plants, such as *A*. *thaliana* and rice, less is known about their function and regulation in other species, including those of agronomic or horticultural value. In this study we used as our experimental system *F*. *vesca*, the wild strawberry, which is diploid, unlike cultivated strawberry (*Fragaria × ananassa*) is octoploid. *F*. *vesca* therefore has a relatively small (~240 Mb) genome, which has been sequenced, as well as a short life cycle (3.5 to 4 months) and a facile transformation system. These characteristics have resulted in a substantial increase in the number of physiological and molecular studies of this species. In cultivated strawberry, only *FaWRKY1* has been characterized to date and it has been shown to be involved in mediating defense responses to the fungus *Colletotrichum acutatum*. Specifically, the expression of *FaWRKY1* is up-regulated in strawberry following *C*. *acutatum* infection, treatments with elicitors, and wounding [31]. However, to our knowledge, there are no reported studies of *WRKY* genes from *F*. *vesca* and nothing is known of their potential association with fruit development and ripening. This is of particular interest given that strawberry is not only an economically important cultivated fruit crop, but also a model system for studies of these processes.

In this current study we identified a total of 59 *FvWRKY* genes from the recently reported *F*. *vesca* ‘Hawaii 4’ genome sequence [32]. We report here the classification, annotation and phylogenetic evaluation of these genes, together with an assessment of conserved motifs and the results of expression profiling of members of the *WRKY* gene family, based on RNA-seq data. The expression patterns of *WRKY* genes in different fruit developmental stages were further investigated using quantitative real-time reverse transcription PCR (qRT-PCR). Our results provide a foundation for further comparative genomics and functional studies of this class of transcriptional regulators in strawberry.

## Materials and Methods

### Identification of Putative *F*. *vesca WRKY* Genes

To generate a comprehensive list of *F*. *vesca WRKY* genes, annotated strawberry protein sequences were downloaded from the public databases, *F*. *vesca* BioView Gene Model Database (https://strawberry.plantandfood.co.nz/) and Genome Database for Rosaceae (http://www.rosaceae.org/species/fragaria/fragaria_vesca/genome_v2.0.a1). The *Arabidopsis WRKY* gene family database was obtained from TAIR (The *Arabidopsis* Information Resource, http://www.arabidopsis.org) and used for comparative analysis.

### Gene Structure Construction, Phylogenetic Analysis and Classification of the *F*. *vesca* WRKY Family

All identified *F*. *vesca WRKY* (*FvWRKY*) genes were classified into different groups based on the *AtWRKY* classification scheme, and the alignment of *FvWRKY* and *AtWRKY* DNA-binding domains was performed using Clustal X 2.147 [33] with default settings. The phylogenetic trees were created using MEGA 5.0 [34] and the neighbor-joining method. Bootstrap values were calculated for 1,000 iterations.

### Analysis of the *FvWRKY* Exon-Intron Structures and Chromosomal Location

The exon-intron organization of the *FvWRKY* genes and their location on strawberry chromosomes were determined based on information available at the National Centre for Biotechnology Information (NCBI; http://www.ncbi.nlm.nih.gov).

### Plant Material and Fruit Pre-Treatments

*F*.*Vesca* (‘Hawaii 4’) were cultivated in growth chamber at 22±1°C in a 13/11 h dark/light photoperiod. Fruit samples were harvested at 18, 24, 30, 36 and 42 DAF (days after flowering). At each developmental stage, ten representative fruits were sampled, snap-frozen in liquid nitrogen and kept at -80°C until further use. For different pre-treatment experiments, two stages (18 and 36 DAF) were selected for sucrose and hormone treatments. The fruits were cut in half longitudinally, and half was used for processing while the other half was used as a control. The hormones used for treatments were indole-3-acetic acid (IAA) and abscisic acid (ABA), each at a concentration of 100 μM and the sucrose concentration used was 50 μM. All experiments were performed at 25°C.

### RNA Extraction, RNA-Seq Based Expression Analysis and Real-Time PCR Analysis

Total RNA was extracted strawberry from ruit harvested at 18, 24, 30, 36 and 42 DAF using the Plant RNA Kit (Omega) according to the manufacturer’s instructions. And Total RNA was reverse transcribed into cDNA by the Invitrogen reverse transcription kit (SuperScript III Reverse Transcriptase). The RNA was subjected to RNA-seq analysis using an Illumina Genome Analyzer at Beijing Novogene Corporation. Real-time PCR was performed using a Light Cycler^®^ 96 SW1.1 Real Time PCR System (Roche), with SYBR-Green (Takara, Dalian, China). The primer sequences used (S1 Table) were designed based on *WRKY* gene sequences using the Beacon designer software. These sequences were subsequently verified using the BLAST tool at NCBI and a dissociation curve was also analyzed after the PCR reaction to confirm their specificity. Each reaction was carried out in a 10 μL volume, consisting of 5 μL SYBR, 3.5 μL ddH_2_O, 1 μL diluted template (1 μL of the generated first-strand cDNA diluted by 9 μL ddH_2_O) and 0.25 μL of each of two gene specific primers. The following program was used for RT-PCR: 95°C for 10 min followed by 40 cycles at 95°C for 20 s, 54°C for 20 s, 72°C for 20 s.

## Results

### Identification and Annotation of the Strawberry WRKY Family and Its Chromosomal Distribution

**Fig 1:**
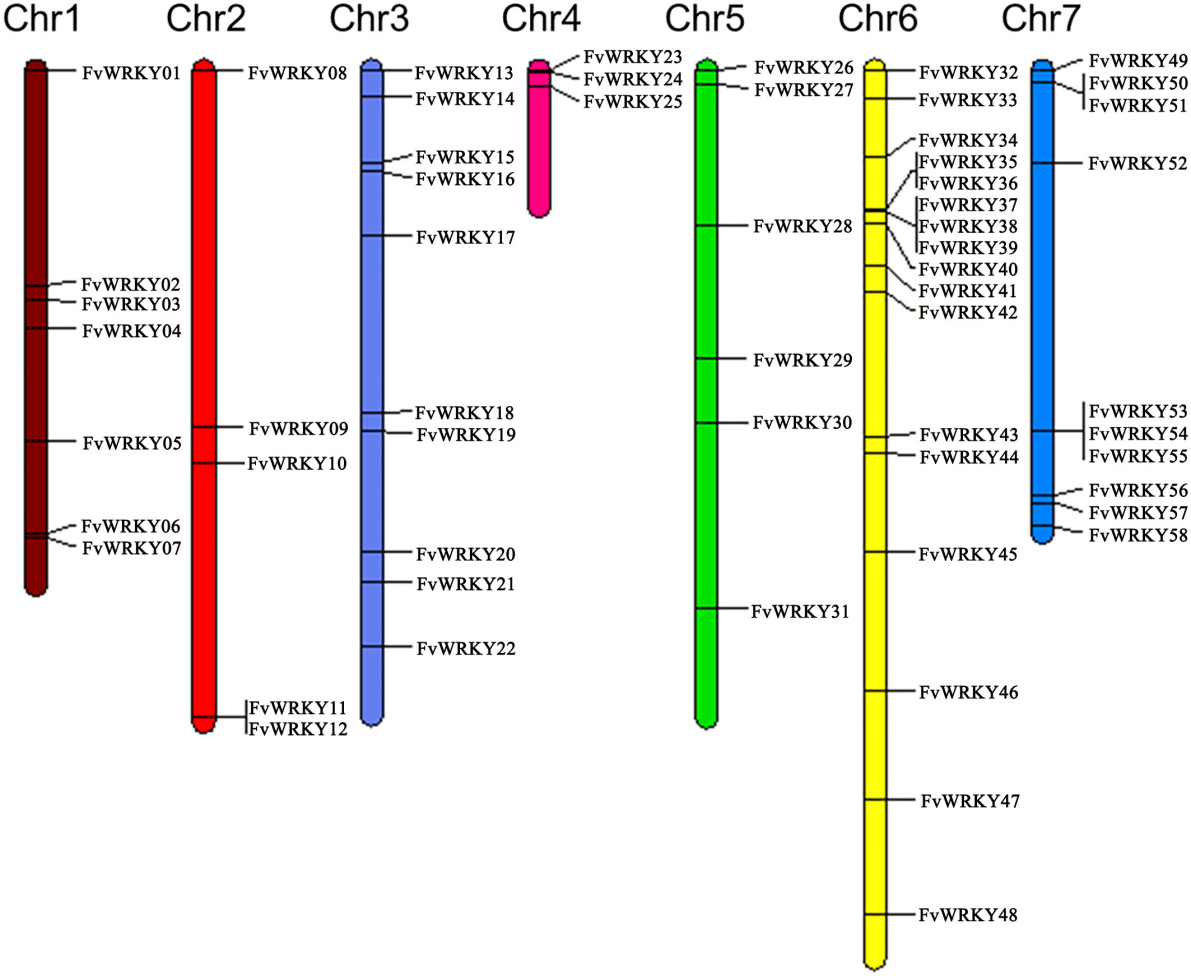
Chromosomal distribution of the *Fragaria vesca* L. *WRKY* gene family. Chromosome size is indicated by relative length. The putative *WRKY* genes from *FvWKRY1* to *FvWRKY58* were renamed based on their placement on the chromosomes. Only one gene, *FvWRKY59*, was not assigned to any chromosome.

A total of 59 full length gene sequences were identified in the strawberry genome as putative members of the *WRKY* superfamily All the 59 gene sequences were obtained from the *F*. *vesca* Whole Genome v2.0.a1 Assembly & Annotation (https://www.rosaceae.org/species/fragaria_vesca/genome_v2.0.a1). All 58 identified *WRKY* genes were mapped to the respective chromosomes and were renamed from *FvWRKY1* to *FvWRKY58*, based on their order on the chromosomes, from chromosomes 1 to 7 (Fig 1) [35]. The parameters used to characterize each of the predicted FvWRKY proteins are listed in Table 1, and included the deduced protein length, molecular weight, isoelectric point, aliphatic index and grand average of hydropathicity. The deduced length of the FvWRKY proteins ranged from 155 (FvWRKY45) to 1,348 amino acids (FvWRKY55), while the pI values ranged from 4.92 (FvWRKY31) to 9.97 (FvWRKY29), which suggests that different FvWRKY proteins might operate in different microenvironments.

**Table 1:** *WRKY* genes identified in *Fragaria vesca*.

Number	Proposed	Gene ID	Chr	ORF (aa)	MW (kDa)	pI	Ai	GRAVY	Instability index (II)	WRKYGQK	Domain pattern	Group	Zinc finger
	name												
1	*FvWRKY01*	101313240	1	467	50.9959	6.68	66	-0.733	58.94	2	C-X_4_-C-X_22_-H-X-H	I	C2H2
2	*FvWRKY02*	101302596	1	600	64.5571	5.2	61.07	-0.713	46.27	1	C-X_5_-C-X_23_-H-X-H	IIb	C2H2
3	*FvWRKY03*	101310739	1	319	34.7101	9.61	62.41	-0.584	52.08	1	C-X_5_-C-X_23_-H-X-H	IId	C2H2
4	*FvWRKY04*	101299520	1	243	27.8051	9.08	60.95	-0.807	54.19	1	C-X_4_-C-X_23_-H-X-H	IIc	C2H2
5	*FvWRKY05*	101295368	1	425	46.6975	6.79	69.39	-0.574	51.93	1	C-X_5_-C-X_23_-H-X-H	IIb	C2H2
6	*FvWRKY06*	101307337	1	317	35.3775	6.66	54.76	-0.747	66.56	1	C-X_4_-C-X_23_-H-X-H	IIc	C2H2
7	*FvWRKY07*	101311038	1	205	22.9693	6.15	47.56	-0.83	42.61	1	C-X_4_-C-X_23_-H-X-H	IIc	C2H2
8	*FvWRKY08*	101300971	2	348	38.908	9.71	67.82	-0.749	56.22	1	C-X_5_-C-X_23_-H-X-H	IId	C2H2
9	*FvWRKY09*	101295677	2	334	36.1088	9.54	58.14	-0.488	52.56	1	C-X_5_-C-X_23_-H-X-H	IId	C2H2
10	*FvWRKY10*	101302035	2	342	37.0599	9.51	62.78	-0.57	51.89	1	C-X_5_-C-X_23_-H-X-H	IId	C2H2
11	*FvWRKY11*	101297319	2	382	42.3033	6.27	62.57	-0.656	54.58	1	C-X_5_-C-X_23_-H-X-H	IIa	C2H2
12	*FvWRKY12*	101297610	2	333	36.8124	8.89	67.69	-0.655	47.47	1	C-X_5_-C-X_23_-H-X-H	IIa	C2H2
13	*FvWRKY13*	101300421	3	571	61.544	7.15	58.37	-0.742	49.19	1	C-X_5_-C-X_23_-H-X-H	IIb	C2H2
14	*FvWRKY14*	101291408	3	505	55.3735	6.4	65.94	-0.917	41.08	2	C-X_4_-C-X_22_-H-X-H	I	C2H2
											C-X_4_-C-X_23_-H-X-H		
15	*FvWRKY15*	101311736	3	355	39.182	7	45.58	-0.943	58.42	1	C-X_4_-C-X_23_-H-X-H	IIc	C2H2
16	*FvWRKY16*	101292382	3	497	53.6208	5.92	52.86	-0.74	51.9	1	C-X_5_-C-X_23_-H-X-H	IIe	C2H2
17	*FvWRKY17*	101314070	3	277	30.4295	5.19	46.86	-0.789	65.15	1	C-X_5_-C-X_23_-H-X-H	IIe	C2H2
18	*FvWRKY18*	101291609	3	547	59.5604	6.22	67.62	-0.59	40.27	1	C-X_5_-C-X_23_-H-X-H	IIb	C2H2
19	*FvWRKY19*	101307469	3	573	62.3768	6.73	54.42	-0.847	57.7	2	C-X_4_-C-X_22_-H-X-H	I	C2H2
											C-X_4_-C-X_23_-H-X-H		
20	*FvWRKY20*	101305151	3	627	68.6763	7.68	42.33	-1.046	49.75	2	C-X_4_-C-X_22_-H-X-H	I	C2H2
											C-X_4_-C-X_23_-H-X-H		
21	*FvWRKY21*	101313293	3	734	79.1599	5.87	51.42	-0.788	51.55	2	C-X_4_-C-X_22_-H-X-H	I	C2H2
											C-X_4_-C-X_23_-H-X-H		
22	*FvWRKY22*	101301660	3	378	41487.2	5.67	45.95	-0.93	58.88	1	C-X_4_-C-X_23_-H-X-H	IIc	C2H2
23	*FvWRKY23*	101292010	4	313	34.1893	6.45	46.04	-0.915	65.87	1	C-X_4_-C-X_23_-H-X-H	IIc	C2H2
24	*FvWRKY24*	101295510	4	499	54.7651	7.69	43.77	-1.029	57.49	2	C-X_4_-C-X_22_-H-X-H	I	C2H2
											C-X_4_-C-X_23_-H-X-H		
25	*FvWRKY25*	101309152	4	615	67.7268	6.67	58.28	-0.812	46.46	1	C-X_5_-C-X_23_-H-X-H	IIb	C2H2
26	*FvWRKY26*	101293392	5	309	35.2252	5.14	58.71	-0.777	61.02	1	C-X_5_-C-X_23_-H-X-H	IIe	C2H2
27	*FvWRKY27*	101310412	5	355	40.0883	4.96	57.15	-0.755	63.99	1	C-X_7_-C-X_23_-H-X-H(C)	III	C2HC
28	*FvWRKY28*	101314661	5	672	72.805	6.95	54.96	-0.834	55.83	1	C-X_5_-C-X_23_-H-X-H	IIb	C2H2
29	*FvWRKY29*	101291727	5	301	32.8982	9.97	57.04	-0.674	53.72	1	C-X_5_-C-X_23_-H-X-H	IId	C2H2
30	*FvWRKY30*	101294370	5	520	57.9652	5.02	57.13	-0.981	60.31	1	C-X_5_-C-X_23_-H-X-H	IIb	C2H2
31	*FvWRKY31*	101296670	5	282	32.3806	4.92	53.19	-1.126	69.82	1	C-X_5_-C-X_23_-H-X-H	IIe	C2H2
32	*FvWRKY32*	101307803	6	319	35.7679	9.58	70.56	-0.641	55.3	1	C-X_5_-C-X_23_-H-X-H	IId	C2H2
33	*FvWRKY33*	101307611	6	727	80.232	5.88	61.49	-0.753	50.05	2	C-X_4_-C-X_23_-H-X-H	I	C2H2
34	*FvWRKY34*	101296583	6	491	53.1033	7.56	64.81	-0.588	56.05	1	C-X_5_-C-X_23_-H-X-H	IIb	C2H2
35	*FvWRKY35*	101295066	6	320	35.7606	5.39	73.78	-0.553	53.99	1	C-X_7_-C-X_23_-H-X-H(C)	III	C2HC
36	*FvWRKY36*	101296502	6	297	33.5437	7.17	69.6	-0.564	51.69	1	C-X_7_-C-X_23_-H-X-H(C)	III	C2HC
37	*FvWRKY37*	101297362	6	231	25.9552	9.32	67.1	-0.659	49.84	1	C-X_7_-C-X_23_-H-X-H(C)	III	C2HC
38	*FvWRKY38*	101297067	6	344	38.7567	5.41	61.77	-0.729	48.9	1	C-X_7_-C-X_23_-H-X-H(C)	III	C2HC
39	*FvWRKY39*	101297653	6	339	38.2152	5.64	58.38	-0.761	46.04	1	C-X_7_-C-X_23_-H-X-H(C)	III	C2HC
40	*FvWRKY40*	101311683	6	517	57.4636	7.63	43.58	-1.016	62.51	2	C-X_4_-C-X_22_-H-X-H	I	C2H2
											C-X_4_-C-X_23_-H-X-H		
41	*FvWRKY41*	101301793	6	296	33.308	5.44	59.26	-0.795	58.73	1	C-X_4_-C-X_23_-H-X-H	NG	C2H2
42	*FvWRKY42*	101296019	6	478	52.1898	9.14	55.46	-0.889	49.8	2	C-X_4_-C-X_22_-H-X-H	I	C2H2
											C-X_4_-C-X_23_-H-X-H		
43	*FvWRKY43*	101301515	6	268	29.9031	5.12	59.25	-0.797	53.14	1	C-X_5_-C-X_23_-H-X-H	IIe	C2H2
44	*FvWRKY44*	101301598	6	326	36.0794	8.8	64.02	-0.789	51.99	1	C-X_5_-C-X_23_-H-X-H	IIa	C2H2
45	*FvWRKY45*	101307027	6	155	17.7763	5.13	43.29	-1.139	49.85	WRKYGKK	C-X_4_-C-X_23_-H-X-H	IIc	C2H2
46	*FvWRKY46*	101308483	6	368	41.6158	6.46	53.83	-0.982	62.25	1	C-X_4_-C-X_23_-H-X-H	IIc	C2H2
47	*FvWRKY47*	101305864	6	519	56652.8	8.57	58.65	-0.84	64.22	2	C-X_4_-C-X_22_-H-X-H	I	C2H2
											C-X_4_-C-X_23_-H-X-H		
48	*FvWRKY48*	101293115	6	213	23.5498	8.76	45.87	-0.982	49.77	1	C-X_4_-C-X_23_-H-X-H	IIc	C2H2
49	*FvWRKY49*	101295163	7	353	39.2606	5.23	56.01	-0.684	49.55	1	C-X_7_-C-X_23_-H-X-H(C)	III	C2HC
50	*FvWRKY50*	101307333	7	226	25.3266	9.16	67.35	-0.781	38.1	1	C-X_4_-C-X_23_-H-X-H	IIc	C2H2
51	*FvWRKY51*	101304921	7	345	37.7218	8.5	51.45	-0.71	49.42	1	C-X_5_-C-X_23_-H-X-H	IIe	C2H2
52	*FvWRKY52*	101312284	7	254	28.6893	7.76	44.84	-1.021	52.76	1	C-X_4_-C-X_23_-H-X-H	IIc	C2H2
53	*FvWRKY53*	101315104	7	379	42.4161	6.63	54.64	-0.731	51.64	1	C-X_7_-C-X_23_-H-X-H(C)	III	C2HC
54	*FvWRKY54*	101306674	7	340	37.927	6.43	53.91	-0.835	49.12	1	C-X_7_-C-X_23_-H-X-H(C)	III	C2HC
55	*FvWRKY55*	101307732	7	1348	152.8182	6.25	91.05	-0.27	42.6	1	C-X_7_-C-X_23_-H-X-H(C)	NG	C2HC
56	*FvWRKY56*	101295568	7	372	40.9578	6.64	66.88	-0.591	53	1	C-X_7_-C-X_23_-H-X-H(C)	III	C2HC
57	*FvWRKY57*	101309009	7	1333	151.6718	6.81	89.56	-0.28	47.5	1	C-X_7_-C-X_23_-H-X-H(C)	NG	C2HC
58	*FvWRKY58*	101304426	7	430	46.3448	5.12	53.77	-0.7	62.01	1	C-X_5_-C-X_23_-H-X-H	IIe	C2H2
59	*FvWRKY59*	101290922	Un	190	21.6712	9.45	48.74	-0.878	41.59	1	C-X_4_-C-X_23_-H-X-H	IIc	C2H2

Abbreviations: Ai, aliphatic index; Chr, chromosome number; GRAVY, grand average of hydropathicity; MW, molecular weight; NG, no chromosomal group identified; ORF, open reading frame; pI, isoelectric point; RDM, random chromosome; UN, unknown chromosome.

Using TblastN, 58 out of the 59 *FvWRKY* genes could be mapped to *F*. *vesca* chromosomes, indicating an unevenly comprehensive distribution of *FvWRKYs* within strawberry genome (Fig 1). One *WRKY* gene (101290922) could not be conclusively mapped to any chromosome and was renamed *FvWRKY59*. Chromosome 6, contained the largest number (17) of *FvWRKY* genes, followed by chromosome 3 and 7, each of which had ten *FvWRKY* genes. Seven were mapped to chromosome 1 and three to chromosome 4. Chromosomes 2 and 5 had five and six *FvWRKY* genes, respectively. According to the definition of a gene cluster provided by Holub [36], twelve *FvWRKY* genes were present in five clusters, of which two were on each of chromosomes 6 and 7, and one cluster was on chromosome 2 (Fig 1).

### *FvWRKY* Exon and Intron Organization

**Fig 2:**
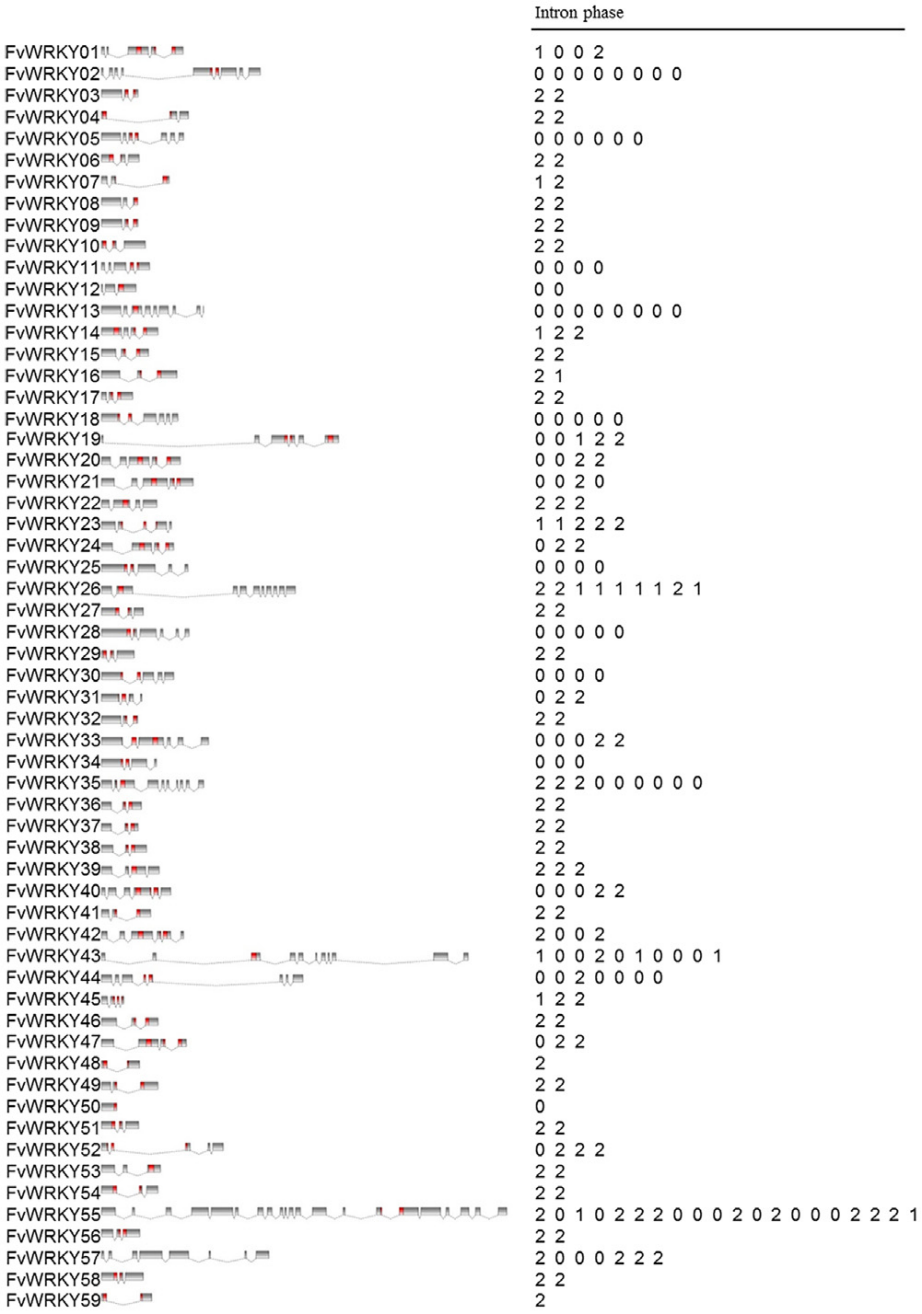
Structures of the *FvWRKY* genes. Gene names are indicated on the left. Exons, represented by black or red boxes, were drawn to scale. Dashed lines connecting two exons represent an intron. Intron phases 0, 1 and 2 are indicated by numbers 0, 1 and 2, respectively. WRKY domains in the corresponding proteins are marked in red.

To investigate the structural evolution of the *F*. *vesca* WRKY family, the exon-intron patterns were analyzed. The number of introns ranged from one to twenty (Fig 2), and a large number of genes contained two introns, while eight genes contained three introns and nine contained four introns. The *FvWRKY44* and *FvWRKY57* genes contained seven introns, *FvWRKY2* and *FvWRKY13* contained eight introns and *FvWRKY26* and *FvWRKY35* contained nine introns. *FvWRKY43* and *FvWRKY55* contained ten and twenty introns, respectively. Two genes contained only one intron and *FvWRKY50* had no introns.

The phylogenetic relationship of the FvWRKY proteins was examined by multiple sequence alignment of their WRKY domains, which were highly conserved (S1 Fig). The intron located in the conserved WRKY domain could be classified as either a R-type intron or a V-type intron. R-type introns are spliced prior to an arginine residue (R), while V-type introns are spliced before a valine residue, located six amino acids after the second cysteine residue of the zinc finger C2H2 motif (S1 Fig). The V-type intron was observed in the WRKY domains of genes belonging to groups IIa and IIb, whereas the R-type intron was widely distributed amongst all other FvWRKY groups (S1 Fig).

### Phylogenetic Analysis, Classification and Motif Analysis of the *F*. *vesca WRKY* Gene Family

**Fig 3:**
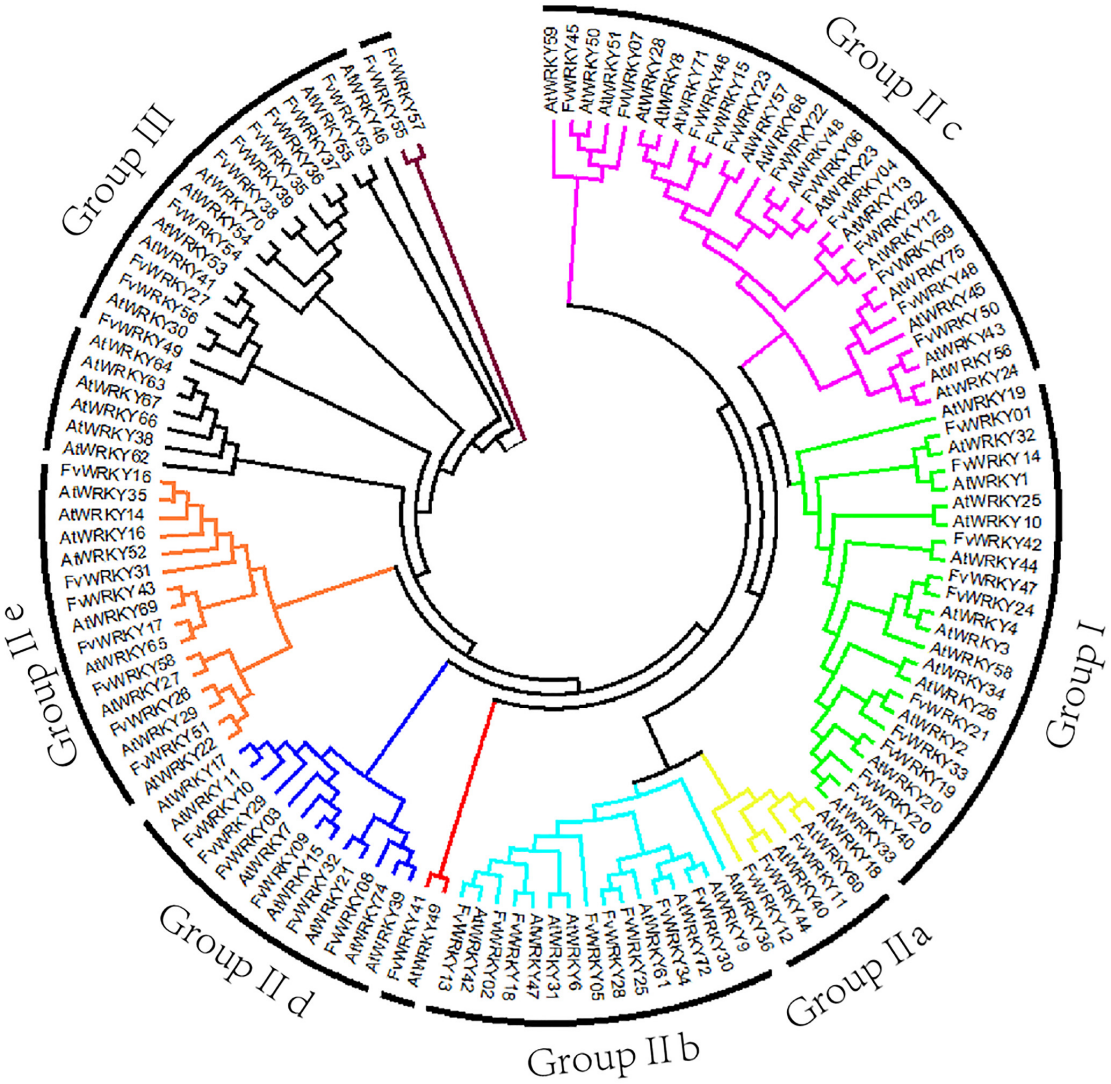
Phylogenetic tree based on WRKY domain sequences from *Fragaria vesca* and *Arabidopsis thaliana*. The phylogenetic tree was created using the MEGA 5.0 software. Reliability of the predicted tree was tested using bootstrapping with 1,000 replicates. Numbers at the nodes indicate how often the group to the right appeared amongst the bootstrap replicates. Branch lines of subtrees are colored indicating different WRKY subgroups.

The distribution of structural domains can provide important insights into the evolution and relationship between highly divergent sequences [36]. Sequence comparisons, as well as phylogenetic and structural analyses showed that the *A*. *thaliana* WRKY domains could be classified into four large groups, termed groups I, II, III and IV [2]. Ten of the FvWRKY proteins belong to Group I, members of which contain two complete WRKY domains and a C_2_H_2_-type zinc finger motif (C-X_4_-C-X_22-23_-H-X_1_-H). Ten FvWRKY proteins, each with a single WRKY domain, were assigned to Group III, which is characterized by a C-X_4_-C-X_23_-H-X_1_-C C_2_HC zinc-finger structure. Finally, thirty-four FvWRKY proteins, possessing a single WRKY domain, were assigned to Group II, members of which differ from Group III FvWRKY proteins based on their C_2_H_2_-type zinc-finger structure (C-X_5_-C-X_23_-H-X_1_-H). Group II could be further subdivided into five distinct subgroups with 3, 8, 12, 6 and 7 members, respectively, based on their primary amino-acid sequence (Group IIa, IIb, IIc, IId and IIe). FvWRKY41 exhibited sequence divergence in the unique WRKY domain and so was not classified into any group. FvWRKY55 and FvWRKY57 were classified into Group IV, since they contained only one WRKY domain and a C_2_HC zinc-finger motif, however, they were distinct from other Group III members (Fig 3).

**Fig 4:**
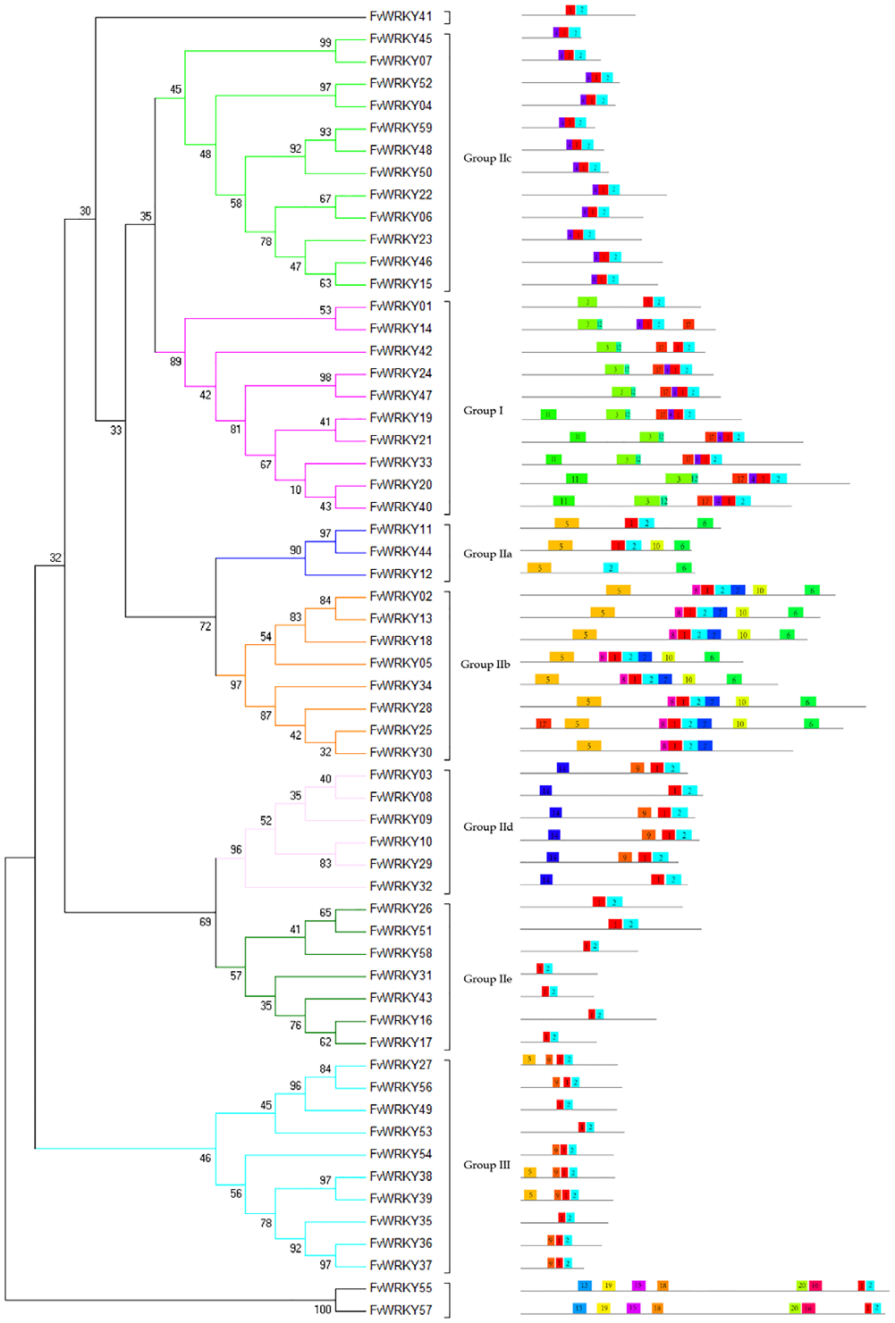
Phylogenetic tree based on the deduced FvWRKY domains and their associated motifs. The phylogenetic tree was constructed using the MEGA 5 software. The reliability of the predicted tree was tested using bootstrapping with 1,000 replicates. Numbers at the nodes indicate how often the group to the right appeared amongst the bootstrap replicates. Subtree branch lines are colored indicating different WRKY subgroups. The motif composition related to each FvWRKY protein is displayed on the right-hand side. The motifs, numbered 1–20, are displayed in different colored boxes. The sequence information for each motif is provided in Table 2.

Conserved motifs other than the WRKY domain were detected by visual inspection (S1 Table). Four members (FvWRKY3, 9, 10 and 29) of Group IId were found to contain the HARF (RTGHARFRR [A/G]P) motif, whose function has not been clearly determined, and a putative leucine zipper structure was detected in FvWRKY11 (Group IIa). The LxxLL co-activator and LxLxLx repressor motifs were found in eight (FvWRKY3, 31, 46, 49, 50, 54, 55 and 57) and thirteen (FvWRKY11, 12, 25, 27, 28, 30, 32, 33, 34, 35, 50, 55 and 57) proteins, respectively, while FvWRKY50, 55 and 57 were observed to have both active repressor and co-activator motifs (Fig 4).

**Table 2:** Analysis and distribution of conserved motifs in *Fragaria vesca* (strawberry) FvWRKY proteins.

Motif	E-value	Width	Best possible match
1	5.0e-1205	25	DGY[RA]WRKYGQK[PV][IV]K[GN][SN]P[YH]PR[SG]YY[RK]C
2	3.4e-994	29	QGCP[VA][RK]K[QH]V[EQ]R[SA]S[ED]DPS[IM][LVF][IV]TTYEGEH[NT][HC]
3	5.6e-314	50	DGYNWRKYGQKQVKGSE[YFN]PRSYY[KR]CT[HF]PNCP[VT]KKKVERS[LH][DE]G[QH]ITEI[IV]YK
4	1.6e-113	15	PR[FV][AV][FV][QM]T[KRT]SE[VI]D[IH]L[DE]
5	4.8e-090	46	ELES[LAV]Q[AE]E[LM][QS][RE][VMG][RN]EE[NA][QK][RKQ]L[RK][GK][MV]LEQ[MTV]T[KE][SDN]Y[NEQ]AL[QE][TM][HKQ][LF]L[DE][VIL][LMR][QT][NQK][EQ]Q[LQ]
6	1.9e-064	29	[LTK][VI][ESAQ][ATQ][AM][TA][AKS][AS][IL]T[AKS]DP[NKT]F[TQ][AST][AV]LAAA[IL][ST]S[IS][IMV]G[NGT]
7	1.1e-061	27	PLP[PM][AS]A[TM][AS]MASTTSAAA[SA]MLLSGS[MS][STP]S
8	7.6e-038	15	[KR]ARVSVRAR[CS][ED][AST]P[MT][IML]
9	6.5e-034	26	KSRE[TS][FS][SK][DG]R[CR][HG][CS][SY]K[KR]RKT[SR][HV][KS][WR]TK[DR]V
10	5.2e-026	25	[CF][GS][SF][SY][MV][AP]T[IL]S[AT]S[AS][PS]FPT[IV]TLDLT[RS]SP
11	3.2e-025	41	[YCHS][LFS][TAY][IL][PV][PN][GA][LFIM][ST]P[TA][ADEST][LF]L[DES]SP[VM][FLM]L[SAP][NS][SM][NKLQ][AIT][LEQ]PSPTTG[SAKT]F[PALS][AFMNS][QLPV][AIKPS][FMNP][NDGV][WGHY]
12	9.6e-019	13	G[EQ]H[ND]H[PA]KPQ[PS][NT][RK][RP]
13	2.0e-018	50	CLG[EV]D[DG]VR[GV][IV]G[IV][CY]GMPGIGKTTIARAVY[DE]EI[TV]CQFEHYCFL[DE]NVKDGFKN
14	4.2e-016	23	[TA]Dx[AT]VSKFKKV[IV]SLLNR[GT][RG][HT][AG][HR][AF]
15	7.1e-016	50	WEDELEKIK[EK]IPH[LM]EIQ[GV]VLRTS[LY][DN]GL[DE]P[LS]QKDIFLDIACFFRGM[DE]KGYV
16	8.3e-015	48	A[IL]C[HY]C[ST][FL]KGNHG[EL]Y[EK]F[NS]F[QT]LLDWGF[RS]T[DN]R[FI]L[EQ]SDHMFL[AG]YVPWS[EQ]YR[CF]
17	1.4e-010	29	[DE][ED][GD][DE][DE][DES]E[PS][ENR][SA]KR[RW][KNR][IKM][ED][GANV]x[AN][SEI]E[VM][AS][AI][PAS][GHT][REKS][ART][IV]
18	1.8e-010	41	WEI[IV]RQQS[IV]K[DE]PGKRSRLW[IV]YEDV[AD][HR]V[FL]TQN[MT]ATDAVE[CG]IM
19	4.0e-010	49	KKVLLVLDDVE[NT]FAQIEALLGKQ[CH]SFGGGSRII[IV]TTRDIQSLSGV[NQ][AE]RY
20	5.5e-010	41	WFN[HN]QC[KR]G[FS]SV[NT]V[KQ]LP[PQ]NWFD[DN][EK]FLGFA[IL]C[AV]VS[DN]FKG[AP]HND

As shown in Table 2, twenty other distinct motifs were identified by the Multiple Expectation Maximization for Motif Elicitation online tool (http://meme-suite.org/tools/meme). The numbers of *FvWRKY* genes in each subgroup were compared with the corresponding numbers in other plant species, i.e., *A*. *thaliana* [37], rice [38], grapevine [22] and cucumber [21] (Table 3). They were found to be generally similar to the distribution in grapevine, although an clear expansion was detected in Group III.

**Table 3:** Size of the WRKY groups and sub-groups in different plant species.

		Group II							
	Group I	IIa	IIb	IIc	IId	IIe	GroupIII	NG	Total
Strawberry	10	3	8	12	6	7	10	3	59
Arabidopsis	16	3	8	17	7	8	14	1	74
Rice	15	4	8	15	7	11	36		96
Grapevine	12	3	8	15	6	7	6	2	59
Cucumber	10	4	4	16	8	7	6		55

Strawberry, *Fragaria vesca*. Arabidopsis, *Arabidopsis thaliana*. Rice, *Oryza sativa*. Grapevine, *Vitis vinifera*, Cucumber, *Cucumis sativus*, NG, no group identified.

### *FvWRKY* Expression Profiles in Different Organs and Fruit Developmental Stages

**Fig 5:**
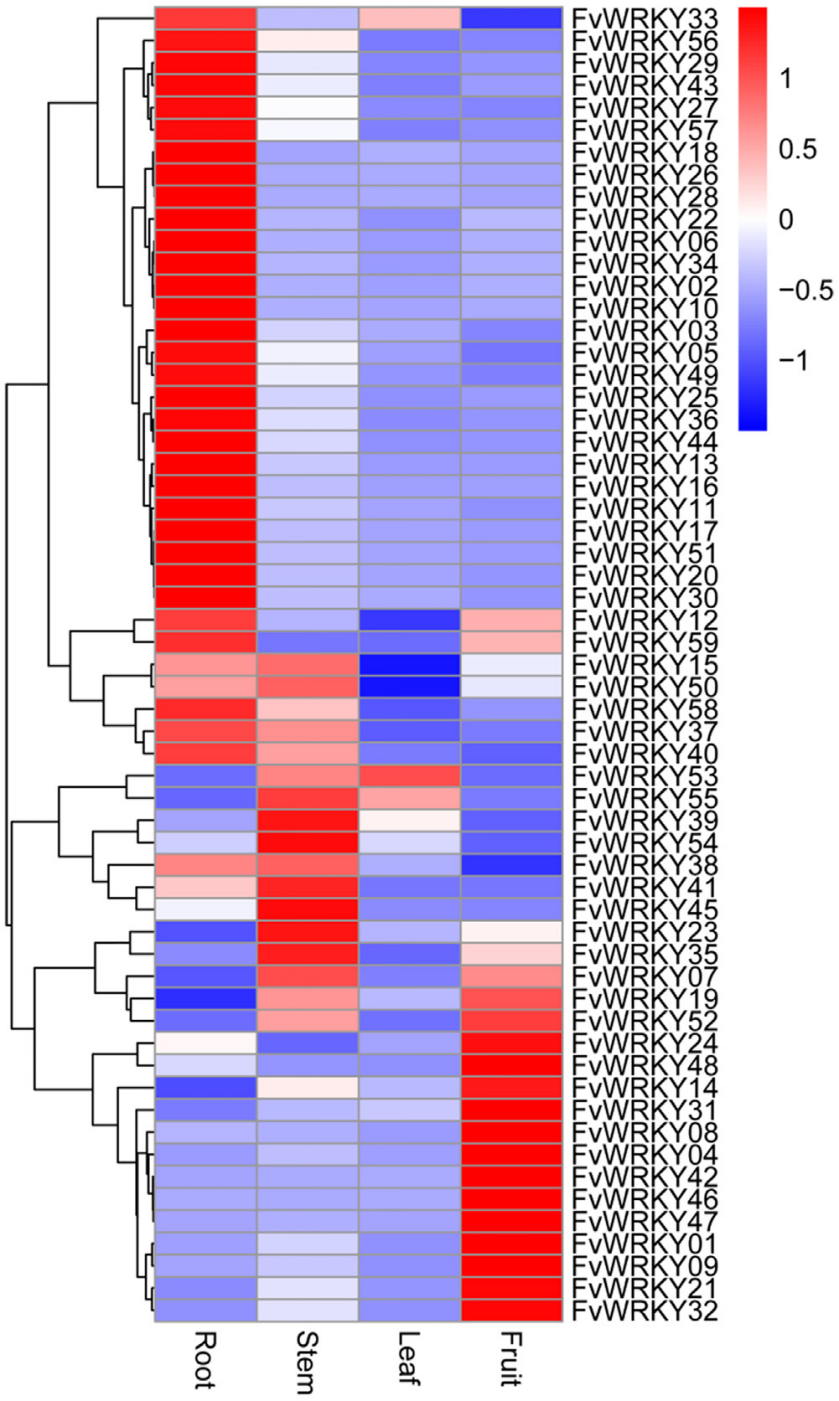
qRT-PCR validation of *FvWRKY* expression in different tissues. Red and blue boxes indicate high and low expression levels, respectively, for each gene.

The expression patterns of all the *FvWRKY* genes were analyzed roots, stems, leaves and fruits of *F*. *vesca* ‘Hawaii 4’ grown under normal conditions. Among the 59 predicted genes, 52 genes were expressed in at least one of the four tissues (Fig 5). Expression of the other seven genes was not detected by real-time RT-PCR; however, it may be that they are expressed in other organs, only expressed in response to specific biotic or abiotic stresses, or are pseudogenes.

**Fig 6:**
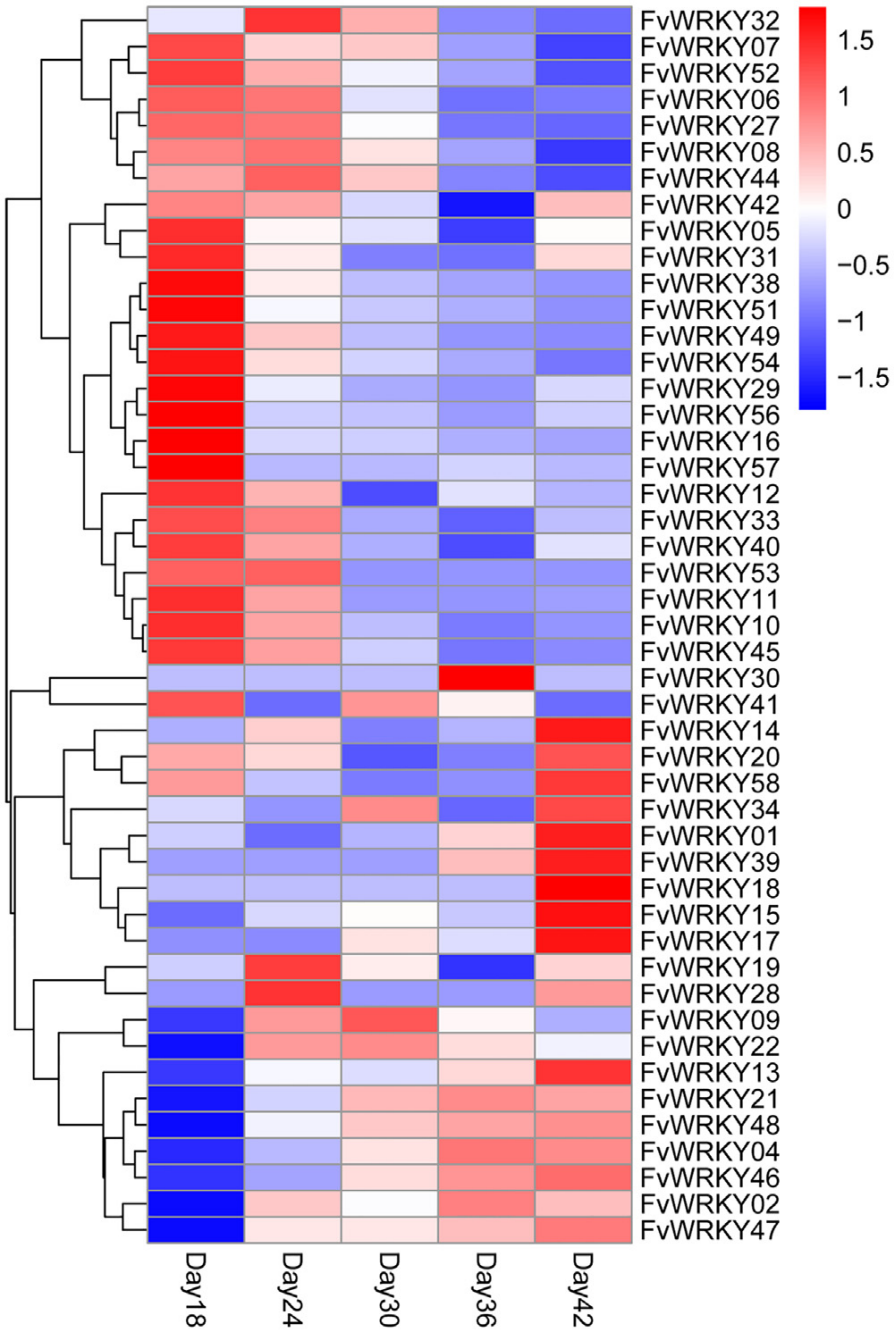
RNA-seq data showing *FvWRKY* gene expression during fruit development and ripening. Red and blue boxes indicate high and low expression levels, respectively, for each gene. Day18, 18 days after flowering; Day24, 24 days after flowering; Day30, 30 days after flowering; Day36, 36 days after flowering; Day42, 42 days after flowering.

**Fig 7:**
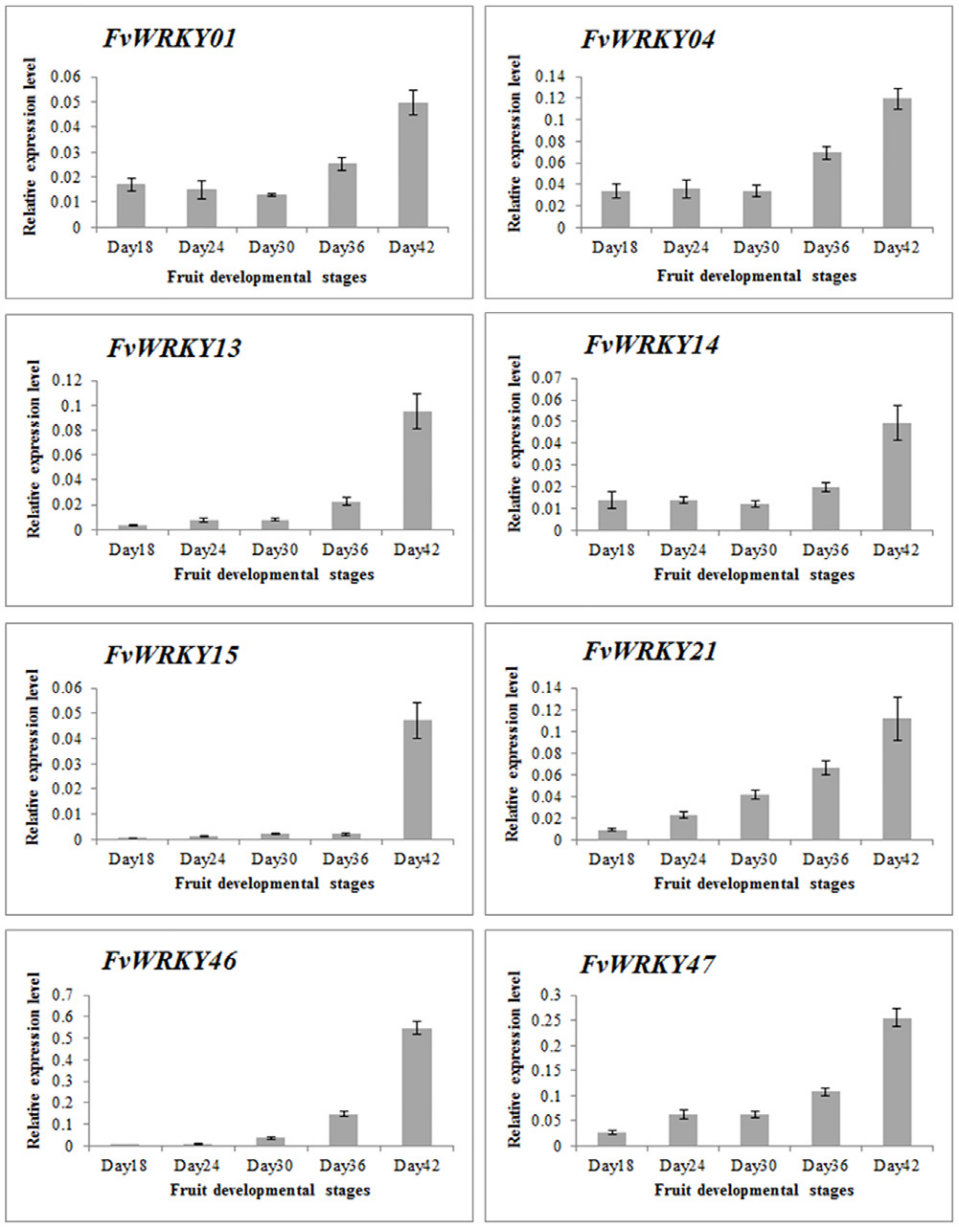
qRT-PCR validation of *FvWRKY* expression during fruit development and ripening. Red and blue boxes indicate high and low expression levels, respectively, for each gene. Day18, 18 days after flowering; Day24, 24 days after flowering; Day30, 30 days after flowering; Day36, 36 days after flowering; Day42, 42 days after flowering.

We next evaluated the expression of the *FvWRKY* genes during fruit development and ripening, based on RNA-seq data. Of the 59 predicted genes, 46 showed expression in fruits (Fig 6), and the expression of *FvWRKY1*, *FvWRKY4*, *FvWRKY13*, *FvWRKY14*, *FvWRKY15*, *FvWRKY21*, *FvWRKY46* and *FvWRKY47* was up-regulated during fruit development and ripening (Fig 7). The greatest increase in expression was observed for *FvWRKY46* and *FvWRKY47* at 42 DAF.

### Exogenous ABA, IAA and Sucrose Induce Accumulation of *FvWRKY* Transcripts in Fruits

**Fig 8:**
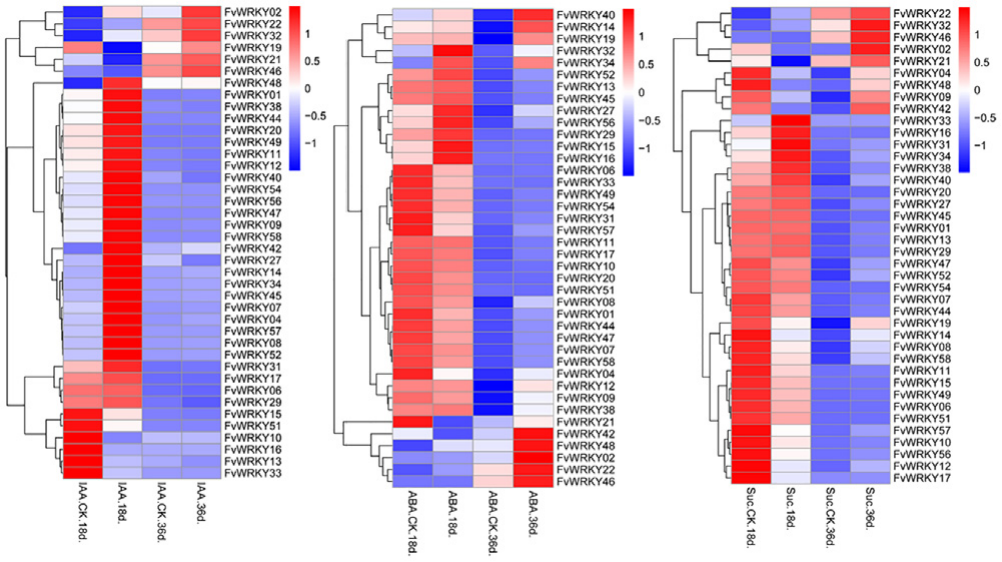
qRT-PCR expression analysis of *FvWRKY* genes in 18 days after flowering (DAF) and 36 DAF fruits treated with IAA, ABA or sucrose. Red and blue boxes indicate high and low expression levels, respectively, for each gene. DAF, days after flowering; IAA.CK.18d., controlled trials of 18 DAF fruits treated with IAA (100 μM); IAA.18d., 18 DAF fruits treated with IAA (100 μM); IAA.CK.36d. controlled trials of 36 DAF fruits treated with IAA (100 μM);IAA.36d., 36 DAF fruits treated with IAA (100 μM); ABA.CK.18d., controlled trials of 18 DAF fruits treated with ABA (100 μM); ABA.18d., 18 DAF fruits treated with ABA (100 μM); ABA.CK.36d. controlled trials of 36 DAF fruits treated with ABA (100 μM);IAA.36d., 36 DAF fruits treated with ABA (100 μM); Suc.CK.18d., controlled trials of 18 DAF fruits treated with sucrose (50 μM); Suc.18d., 18 DAF fruits treated with sucrose (50 μM); Suc.CK.36d. controlled trials of 36 DAF fruits treated with sucrose (50 μM); Suc.36d., 36 DAF fruits treated with sucrose (50 μM).

We used qRT-PCR analysis to examine the expression of the *FvWRKY* genes in 18 DAF and 36 DAF fruits in response to exogenous IAA, ABA and sucrose. After IAA treatment, most *WRKY* genes were expressed in the early stages of fruit development. Thirty-four genes were detected in 18 DAF fruit and the expression of *WRKY02*, *19*, *21*, *22*, *32* and *48* was induced in 36 DAF fruits (Fig 8). Addition of ABA, which is known to regulate fruit development and ripening [39], resulted in similar expression patterns as those resulting from the sucrose treatment (Fig 8). The expression levels of most of the *WRKY* genes was reduced by the sucrose treatment and expression was only be detected in 18 DAF fruits, while only ten *WRKY* genes were expressed in 36 DAF fruits and were also induced by sucrose (Fig 8).

## Discussion

### Organization of the *F*. *vesca WRKY* Gene Family

The size of the *F*. *vesca WRKY* gene family (59; genome size 240 Mb) is smaller than those of *A*. *thaliana* (72, genome size 125 Mb) and rice (96; genome size 480 Mb). When the number of *WRKY* genes in the different subgroups was further compared with those of *A*. *thaliana*, rice, grapevine, cucumber and *F*. *vesca* (Table 3), we found that the numbers of *WRKY* genes in Group I, Group IIc and III showed the greatest diferrences between *F*. *vesca* and *A*. *thaliana* and rice, but that there were similar percentages of genes in Group I of *F*. *vesca*, cucumber and grapevine. It has been reported that 80% of the rice *WRKY* gene loci are located in duplicated regions and that gene duplication events have lead to the generation of new *WRKY* genes [20]. Compared with cucumber (genome size ~367 Mb) [21] and grapevine (genome size ~400 Mb) [22], the size of the *WRKY* family in *F*. *vesca* is similar (55 and 59 *WRKY* genes in cucumber and grapevine, respectively); however *F*. *vesca* contain fewer genes in Group IIc and more in Group III.

The structure of the phylogenetic tree based on an alignment of the WRKY domains of *F*. *vesca* and *A*. *thaliana* indicated that the 59 *FvWRKY* proteins can be divided into three major groups (I, II and III) as previously described for other plant species [2] together with one smaller group (IV). Members within the same group or subgroup within group II shared a similar gene structure (intron/exon organization), length and amino-acid motif composition, indicating their close evolutionary relationship.

Group I WRKY proteins, with two WRKY domains, constitute approximately 20% of the entire FvWRKY family, which s comparable in size to those of cucumber and grapevine, but smaller than those of *A*. *thaliana* and rice (Table 3). A significant expansion exists in poplar (*Populus tremula)*, where Group I contains approximately 50% of the entire PtWRKY family. The subgroups of Group II in *F*. *vesca*, *A*. *thaliana*, rice and grapevine are similar in size, except that there is a reduction in Group IIc in *F*. *vesca*. In *F*. *vesca*, the members of Group IIa and IIb are characterized by the presence of motifs 5 and 6, whereas Group IId contains the HARF sequence (motif 14), as described in *A*. *thaliana* [2]. Group III proteins with the C-X_7_-C-X_23_-H-X_1_-H domain pattern, were found to be smaller than in *A*. *thaliana* and even more so than in rice, but larger than in grapevine and cucumber. In the phylogenetic tree, group III was most closely related to the large subgroup IIe+d. A search of the Plant Transcription Factor Database showed that the earliest known evolutionary occurrence of group III genes was in the lycophyte, *Selaginella moellendorffii*. There were also no evidence of any sequenced plant species that contain only members of group I and III but some species have only members of group I and II, such as the moss, *Physcomitrella patens* [5]. We therefore propose that group III may have evolved from group II, as exemplified by group IIe of strawberry. Gene orthology can provide a starting point for genetic studies that aim to define the function of a candidate gene. In the *F*. *vesca* genome, both the *FvWRKY55* and *FvWRKY57* genes contain the domain pattern that is typical of group III, although it has a motif that is not present in other group III members, so we classified these two *WRKY* genes into a small group IV (Fig 4). The biological functions of group IV WRKY genes remain to remains to be determined.

Gene duplication and divergence events have been suggested to be the main contributors to evolutionary momentum. The current gene duplication analysis indicated that 12 of 59 *FvWRKY* genes were associated with either tandem or segmental duplication events and three pairs of the *WRKY* genes (*FvWRKY11/FvWRKY12*, *FvWRKY35/FvWRKY36* and *FvWRKY38/ FvWRKY39*) appeared to have undergone tandem duplication (Fig 4). Our data suggest a low frequency of tandem and a high frequency of segmental duplication events exists amongst the *FvWRKY* genes, which is consistent with results from *A*. *thaliana*. It has been noted that segmental duplication may more often be retained due to sub-functionalization, without increasing the likelihood of gene rearrangement.

### *FvWRKY* Genes Involved in Growth and Development

We evaluated the expression patterns of all of the predicted coding members of the *FvWRKY* family in different strawberry organs at various developmental stages. The data revealed that many of the genes could be grouped together based on their abundant expression in specific organs, possibly reflecting their involvement in a common metabolic and/or developmental process. Indeed, increasing numbers of studies suggest a role for *WRKY* genes in developmental processes, including seed and trichome development, dormancy and germination, and senescence [7, 30, 40–45]. The expression analysis revealed that *FvWRKY4*, *46* and *48* of Group IIc were highly expressed in fruits and that their expression increased during fruit development, suggesting a putative role in the regulation of fruit development and ripening. Involvement of *WRKY* genes within same group in the same developmental processes has been reported previously in both grapevine and *A*. *thaliana* [40, 44, 45]. In *A*. *thaliana*, *AtWRKY6* (Group IIb), has been shown to be strongly upregulated during leaf senescence and to target a senescence-specific receptor-like kinase (SIRK/FRK1) [46]. Other *A*. *thaliana WRKY* genes are also upregulated in the leaf transcriptome during senescence, and *AtWRKY53* and *AtWRKY70* are known to regulate leaf senescence [47–49]. In this current study, the expression of only *FvWRKY53* and *FvWRKY55* (Group III) was detected in leaves, while most genes were expressed in roots and fruits.

### Addition of Exogenous ABA, IAA and Sucrose Induces *FvWRKY* Expression

We investigated the interaction between hormone and metabolite signaling pathways and *FvWRKY* gene expression patterns in fruit. Almost all the *FvWRKY* genes were expressed in fruit that had been treated with exogenous ABA and IAA. In early stages of fruit development, most *FvWRKY* genes were induced by the IAA treatment, but only a few by ABA. Sucrose treatment was seen to inhibit the expression of most *FvWRKY* genes in fruit. Only the expression of *FvWRKY16*, *FvWRKY31*, *FvWRKY33* and *FvWRKY34* were induced by sucrose in the early stages of fruit development, while the expression of *FvWRKY2*, *FvWRKY21*, *FvWRKY22*, *FvWRKY32* and *FvWRKY46* was induced by sucrose in later fruit developmental stages. We observed that several *FvWRKY* genes that were induced by ABA, IAA and sucrose showed the same expression patterns in the late fruit development stage, notably *FvWRKY2*, *FvWRKY22* and *FvWRKY46*. This suggests that these three *FvWRKY* may share the ABA, IAA and sucrose signaling network.

## Conclusions

In the present study, identified total of 59 *F*. *vesca WRKY* genes and characterized their expression profiles in different organs and fruit developmental stages. Among the 59 predicted genes, 52 genes were expressed in at least one of four organs. qRT-PCR analyses were used to assess the expression of *FvWRKY* genes in 18 DAF and 36 DAF fruits in response to exogenous IAA, ABA and sucrose treatments. This analysis suggested that some *FvWRKY* genes may operate in the same ABA, IAA and sucrose signaling network. Our results also indicate that positive selection may have driven the functional divergence of duplicated genes during the expansion of group III *WRKY* genes. Based on the results presented here, we propose that a subset of WRKY proteins contribute to the regulation of strawberry fruit development and ripening.

## Supporting Information

S1 Data (XLS)

S1 Fig Alignment of the amino acid sequences of multiple FvWRKY domains. (TIF)

S1 Table Primer sequence information. This file lists the primer sequences used for real-time quantitative RT-PCR validation of the RNA-seq data.
(DOC)
