# Supplementary figures and images for: Genome-Wide Analysis of the Expression of WRKY Family Genes in Different Developmental Stages of Wild Strawberry (Fragaria vesca) Fruit
Source: PLoS One. 2016 May 3;11(5):e0154312. doi: 10.1371/journal.pone.0154312 (PMC4854424; doi:10.1371/journal.pone.0154312)

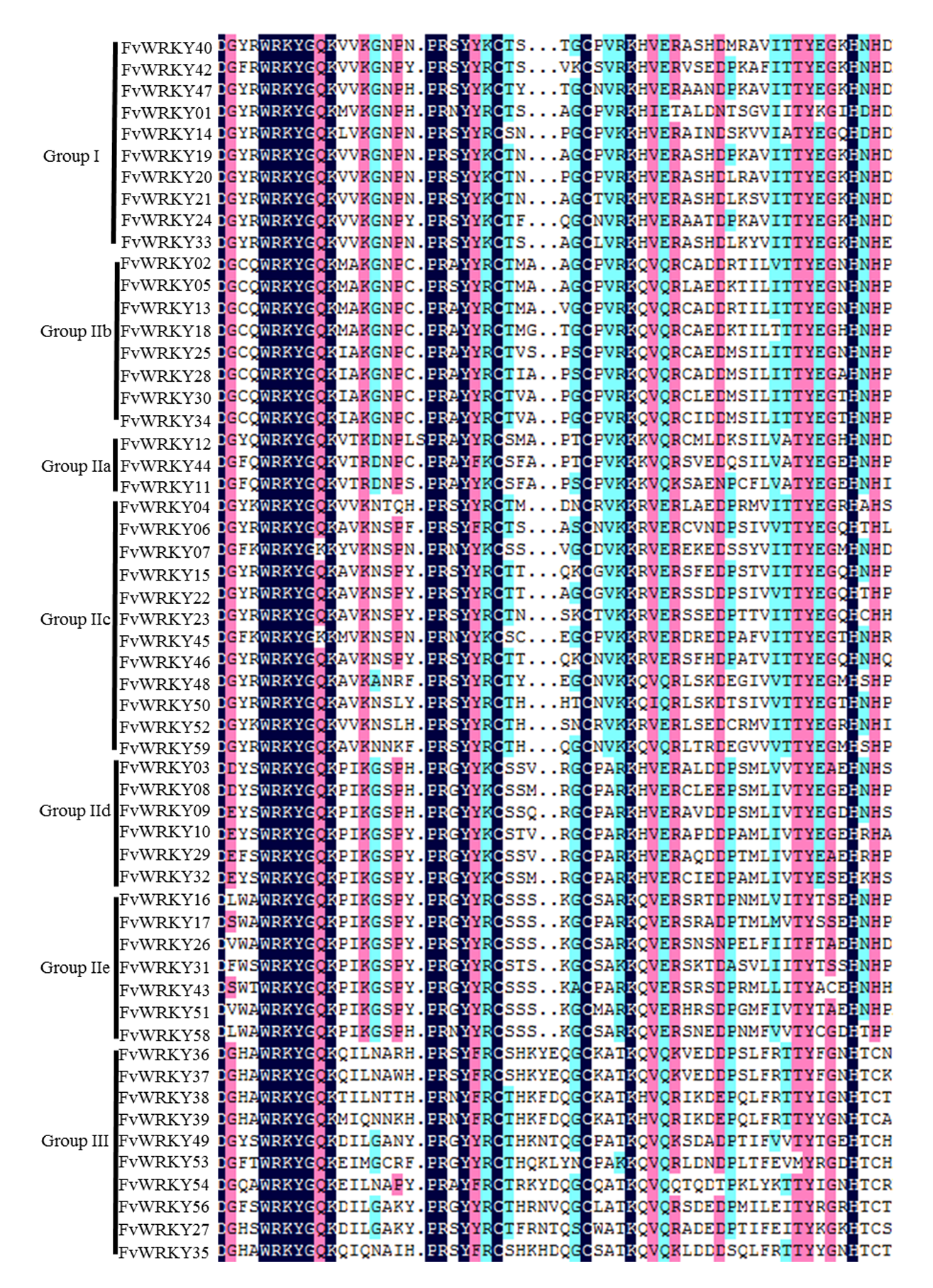

Supplement: S1 Fig — (TIF) [file pone.0154312.s002.tif]
