# Supplementary material for: Genome-Wide Analysis of the Expression of WRKY Family Genes in Different Developmental Stages of Wild Strawberry (Fragaria vesca) Fruit
Source: PLoS One. 2016 May 3;11(5):e0154312. doi: 10.1371/journal.pone.0154312 (PMC4854424; doi:10.1371/journal.pone.0154312)
Supplement: S1 Table — This file lists the primer sequences used for real-time quantitative RT-PCR validation of the RNA-seq data. (DOC) [file pone.0154312.s003.doc]

S1 Table. Primer sequence information.

| Gene | Sequences 5'→3' | Annealing Temperature |
| --- | --- | --- |
| *FvWRKY01* | ATAAGCCACCAGCAAGTTCT | 54^o^C |
|  | TCGCCAGTTGTAACCATCC |  |
| *FvWRKY02* | CCACCTCTACCACCTACCA | 54^o^C |
|  | CATCATCCACAACCGACTGA |  |
| *FvWRKY03* | TTCGTCATCTTCTTCGTCATCC | 54^o^C |
|  | CGTAGCAGCGGAGGAGTA |  |
| *FvWRKY04* | AACCCTAATCCCATCTCTCAATAC | 54^o^C |
|  | GCACATCAACTTCGCTCATT |  |
| *FvWRKY05* | TGAGTTACAGCGAGAACACAA | 54^o^C |
|  | CTTCCTCCACTGACATCCATC |  |
| *FvWRKY06* | CTGCCGACCACTTCCATT | 54^o^C |
|  | TCTCCATCTGTATCCATCTTCCA |  |
| *FvWRKY07* | GATTCGTCGTCGCTGATGA | 54^o^C |
|  | TTCTTTCTCCCTCTCTACTCTCTT |  |
| *FvWRKY08* | GTGGTAGCAGTGGTAGATGTC | 54^o^C |
|  | TCGTTGTGGATTGTGATGGTAT |  |
| *FvWRKY09* | TACCACAACAAGCACCACAA | 54^o^C |
|  | TGAACGAAGAAGAAGGAAGAGG |  |
| *FvWRKY10* | TGGCGGTGGAGATGATGA | 54^o^C |
|  | GAGGCGGAGGAGGAAGAG |  |
| *FvWRKY11* | GGCTTATGTTCGCTGTGATG | 54^o^C |
|  | GTAGGACTTGAAGATGGTGATGA |  |
| *FvWRKY12* | CTGCTCCGACTCCAACTTC | 54^o^C |
|  | ACCTTCTGTCCGTACTTCCT |  |
| *FvWRKY13* | CTGGCACTGTTACAAGTTGAG | 54^o^C |
|  | GAGTTCTGGCTTCGGATGA |  |
| *FvWRKY14* | CATTGCTGACACCTGAGACT | 54^o^C |
|  | CTCTTCACCAACGGACACA |  |
| *FvWRKY15* | AACTCCTCGGTATCTTCCTCTT | 54^o^C |
|  | GCTTCTTGGGTATGGGCTATT |  |
| *FvWRKY16* | CTGGTGGTGTTATGGTTGGA | 54^o^C |
|  | CGGCTACGCTCTACTTGTT |  |
| *FvWRKY17* | CACTCCGCCGTCTGATTC | 54^o^C |
|  | TCTCCTCCTCCTCCTCCT |  |
| *FvWRKY18* | AAGTATGGTCAGAAGATGGCTAA | 54^o^C |
|  | GCAGTTGTGGATGATGAAGAG |  |
| *FvWRKY19* | AAGTCCAAGGTCAAGGTCAAG | 54^o^C |
|  | CCAGTTATAGCCATCATCAGAAGA |  |
| *FvWRKY20* | CTTCTCCGACCTCCTATCCT | 54^o^C |
|  | CTCTGGCTGTTGTTGTTATTGTT |  |
| *FvWRKY21* | CTTGGCGAATCAGGCTCTT | 54^o^C |
|  | CTTCTTCCTCTGGTTGCTCAT |  |
| *FvWRKY22* | CTGGTTGCGGTGTGAAGA | 54^o^C |
|  | TGACGAGGTGGTGGTAGTAA |  |
| *FvWRKY23* | GAGGATGGCTACCGTTGG | 54^o^C |
|  | TGAACTGTGAGACTGGAGGA |  |
| *FvWRKY24* | CATCAGCGTCCTCAGTCTAAG | 54^o^C |
|  | GTCTCTTGGCATCAGGTTCA |  |
| *FvWRKY25* | TTCGTCATCGTCTTCCTCTTC | 54^o^C |
|  | CGCCGCTAATGCTGATTG |  |
| *FvWRKY26* | GCAAGCAGTAGTGAGAGGAT | 54^o^C |
|  | GCATATCGGTGAGGATTCTTTC |  |
| *FvWRKY27* | CTCAATCGGCTGTGCTTCA | 54^o^C |
|  | GTTGGGTCTTCATCTGCTCTC |  |
| *FvWRKY28* | GATGAAGCAGGCGAGACA | 54^o^C |
|  | CGTGGTGATTAGGATGGACAT |  |
| *FvWRKY29* | GCCTCCGCTCTCACAATC | 54^o^C |
|  | GCTCGGCTTCGTAGGTAAC |  |
| *FvWRKY30* | GTCCATACTGATAACAACCTACGA | 54^o^C |
|  | ATGATGATGAGGCTGATGATGAA |  |
| *FvWRKY31* | TGCTCATAATCACCTACACTTCTA | 54^o^C |
|  | TCTTGCTCTTGTTCTTGTTCTG |  |
| *FvWRKY32* | TGGTCGTGTGGCTAACTATG | 54^o^C |
|  | TGCTACATTCTTACCTCCTTCAG |  |
| *FvWRKY33* | GGATTCAATGCCGAGAGGAT | 54^o^C |
|  | GAACTGTCACCATTAGGAGGAA |  |
| *FvWRKY34* | GACCTCACATCTTCTCCTTCC | 54^o^C |
|  | GCACCACCTCCAACCATAG |  |
| *FvWRKY35* | CTACAAGCACTACTCCAACAGAA | 54^o^C |
|  | CGAACACCACCGTATCAGG |  |
| *FvWRKY36* | TGAAGATGATCCGTCGTTGT | 54^o^C |
|  | AGTTCGTAAGGTAAGTTGTTGTTC |  |
| *FvWRKY37* | GGTAGTCGGTAATAGTGATGGT | 54^o^C |
|  | TCTTGATCGTCTTGAGAGCAT |  |
| *FvWRKY38* | GATTGTTCAAGTCGTCAAGAGA | 54^o^C |
|  | TCGGTTGTGGTTGGTCAT |  |
| *FvWRKY39* | TGTGTCGTCCAACCAGAAG | 54^o^C |
|  | ACCAGAACCAGAAGCAGAAG |  |
| *FvWRKY40* | CTCTCCTCCTCCTCCTTCC | 54^o^C |
|  | TCCTTCTCCGCTCCTTGT |  |
| *FvWRKY41* | GACCACCACGAGCAAGTAG | 54^o^C |
|  | ACGGTAAGAGCAACAAGAAGAA |  |
| *FvWRKY42* | CTTGTGGAGTTAGCGGAGAAT | 54^o^C |
|  | CGGAAGCCATCACCTGTAAT |  |
| *FvWRKY43* | CGACATCACCCACCAAAGA | 54^o^C |
|  | AACCTCATTGCCACATCCAT |  |
| *FvWRKY44* | CAACAATAGTGGTAACAACATCCT | 54^o^C |
|  | GGTCTCTTCTCTTGGCTTCT |  |
| *FvWRKY45* | TCCAGCCATCCAGACTTC | 54^o^C |
|  | CTTCCACCAGAATCATCATCATT |  |
| *FvWRKY46* | GGTGCTGCTCGTCATCAT | 54^o^C |
|  | TTCTCCATCTGTAGCCATCTTC |  |
| *FvWRKY47* | GATGATGGTTACAGGTGGAGAA | 54^o^C |
|  | TGAGGCGTTATTGGCTATGTT |  |
| *FvWRKY48* | GCTTCATCCTCTTCGTCAAT | 54^o^C |
|  | GTCATCACCGCCATAGTTG |  |
| *FvWRKY49* | CCTTCCACGCCAATAGAGT | 54^o^C |
|  | AAGCCGACAGCATCTCAT |  |
| *FvWRKY50* | ATCCTCCTCCTCTTCCTCTTC | 54^o^C |
|  | TCATCACCCTCAATAGCATTCTC |  |
| *FvWRKY51* | CACCAACTCACCGCAACT | 54^o^C |
|  | TAACGCTCCGACCAACCT |  |
| *FvWRKY52* | AGCAGTCTTCTTCTTCTCATCATC | 54^o^C |
|  | TGCCTGGTCGTTGTTATTCC |  |
| *FvWRKY53* | GAGTAGTGCTGCGGATTCA | 54^o^C |
|  | CCTTCTTCGTGGTCTGCTT |  |
| *FvWRKY54* | ACGGACAACCTTAACGATACA | 54^o^C |
|  | AATACAGTAGTCTCAGCCTCAG |  |
| *FvWRKY55* | CATCAACGAAGAAGCAGTCA | 54^o^C |
|  | AATCCATTCCAACCAAGTCATT |  |
| *FvWRKY56* | CGAGGAAGGCACACTTGTA | 54^o^C |
|  | TATGTTGAGGCTGGTGATTGA |  |
| *FvWRKY57* | GCGGCATTCACGACTACT | 54^o^C |
|  | TCCTCATCCTCCTCATCTTCTT |  |
| *FvWRKY58* | GCTGTCCGTTGTTGTGTTC | 54^o^C |
|  | CTCCGCACCTCTTCTACTTC |  |
| *FvWRKY59* | AAGCAGTCAAGAACAACAAGT | 54^o^C |
|  | GTCACCACAACTCCTTCATC |  |
